# Molecular Evolution of Porcine Reproductive and Respiratory Syndrome Virus Field Strains from Two Swine Production Systems in the Midwestern United States from 2001 to 2020

**DOI:** 10.1128/spectrum.02634-21

**Published:** 2022-05-02

**Authors:** Ruwini Rupasinghe, Kyuyoung Lee, Xin Liu, Phillip C. Gauger, Jianqiang Zhang, Beatriz Martínez-López

**Affiliations:** a Center for Animal Disease Modeling and Surveillance (CADMS), Department of Medicine and Epidemiology, School of Veterinary Medicine, University of California, Davis, California, USA; b Department of Computer Science, University of California, Davis, California, USA; c Department of Veterinary Diagnostic and Production Animal Medicine, College of Veterinary Medicine, Iowa State University, Ames, Iowa, USA; City University of Hong Kong

**Keywords:** PRRSV-2, wild-type, MLV, RFLP, N-glycosylation, selection pressure

## Abstract

Porcine reproductive and respiratory syndrome virus (PRRSV) poses an extensive economic threat to the United States swine industry. The high degree of PRRSV genetic and antigenic variability challenges existing vaccination programs. We evaluated the ORF5 sequence of 1,931 PRRSV-2 strains detected from >300 farms managed by two pork production systems in the midwestern United States from 2001 to 2020 to assess the genetic diversity and molecular characteristics of heterologous PRRSV-2 strains. Phylogenetic analysis was performed on ORF5 sequences and classified using the global PRRSV classification system. N-glycosylation and the global and local selection pressure in the putative GP5 encoded by ORF5 were estimated. The PRRSV-2 sequences were classified into lineage 5 (L5; *n* = 438[22.7%]) or lineage 1 (L1; *n* = 1,493[77.3%]). The L1 strains belonged to one of three subclades: L1A (*n* = 1,225[63.4%]), L1B (*n* = 69[3.6%]), and L1C/D (*n* = 199[10.3%]). 10 N-glycosylation sites were predicted, and positions N44 and N51 were detected in most GP5 sequences (*n* = 1,801[93.3%]). Clade-specific N-glycosylation sites were observed: 57th in L1A, 33rd in L1B, 30th and 34th in L1C/D, and 30th and 33rd in L5. We identified nine and 19 sites in GP5 under significant positive selection in L5 and L1, respectively. The 13th, 151st, and 200th positive selection sites were exclusive to L5. Heterogeneity of N-glycosylation and positive selection sites may contribute to varying the evolutionary processes of PRRSV-2 strains circulating in these swine production systems. L1A and L5 strains denoted excellence in adaptation to the current swine population by their extensive positive selection sites with higher site-specific selection pressure.

**IMPORTANCE** Porcine reproductive and respiratory syndrome virus (PRRSV) is known for its high genetic and antigenic variability. In this study, we evaluated the ORF5 sequences of PRRSV-2 strains circulating in two swine production systems in the midwestern United States from 2001 to 2020. All the field strains were classified into four major groups based on genetic relatedness, where one group is closely related to the Ingelvac PRRS MLV strain. Here, we systematically compared differences in the ORF5 polymorphisms, N-glycosylation sites, and local and global evolutionary dynamics between different groups. Sites 44 and 51 were common for N-glycosylation in most amino acid sequences (*n* = 1,801, 93.3%). We identified that the L5 sequences had more positive selection pressure compared to the L1 strains. Our findings will provide valuable insights into the evolutionary mechanisms of PRRSV-2 and these molecular changes may lead to suboptimal effectiveness of Ingelvac PRRS MLV vaccine.

## INTRODUCTION

Porcine reproductive and respiratory syndrome (PRRS) is one of the most common and economically significant infectious diseases that affect the global swine industry. This syndrome is caused by the PRRS virus (PRRSV), a small, enveloped, non-segmented, positive-sense, single-stranded RNA virus in the genus *Betaarterivirus* in the family *Arteriviridae*, and primarily associated with reproductive impairment or breeding failures in sows and respiratory disease in pigs of any age (1, 2). PRRSV was first identified in the United States in 1987 and currently exists in both endemic and epidemic forms in the United States’ swine production systems (3). According to Tousignant et al., up to 40% of breeding herds in the United States get PRRS outbreaks annually (4). Therefore, PRRS imposes a significant financial burden on swine producers in the United States, where the annual economic losses of PRRS were estimated at around $664 million in 2013 (5). Various control measures are currently utilized to prevent and eliminate the PRRS, such as implementing efficient herd management strategies, improving biosecurity measures, and executing proper immunization. Nevertheless, PRRSV continues to circulate and evolve, challenging the control programs worldwide.

Currently, two species within the genus *Betaarterivirus* are recognized: *Betaarterivirus suid 1* (PRRSV-1 [European] virus) and *Betaarterivirus suid 2* (PRRSV-2 [North American] virus), which were previously considered to be two genotypes of the PRRSV species (1, 6, 7). Despite the two species causing similar disease syndromes in pigs, they exhibited high genetic diversity, sharing around 65% identity at the nucleotide level (8). Additionally, a greater degree of genetic variation exists within each species (9). So far, PRRSV-2 has been classified into nine distinct lineages based on a comprehensive study of ORF5 sequences, and most of them were identified among swine herds in the United States (10–13). PRRSV-2 genome contains 10 open reading frames (ORF): 1a, 1b, 2a, 2b, 3, 4, 5, 5a, 6, and 7 (14–16). ORF5 encodes the GP5 protein, which is the most crucial immunogenic protein in PRRSV-2 due to the ubiquity of requisite structural entities, including epitopes, hypervariable regions, and N-linked glycosylation sites (17, 18). Two epitopes, epitope A (a neutralizing epitope [NE] comprises amino acids 37 to 41), and epitope B (a decoy epitope [DE] involving amino acids 27 to 31) (19–21), and two hypervariable regions, HVR1 (32 to 36 amino acids) and HVR2 (57 to 61 amino acids) (21, 22) have been discovered in the GP5 of PRRSV-2 (reference strain: VR-2332). They are presumably responsible for substantially high genetic and antigenic diversity within each species, host immune evasion, and partial-cross protection elicited by the current vaccines (22, 23). Further, the deletion or insertion of N-linked glycosylation sites in GP5 alters the viral susceptibility to the host’s neutralizing antibody through the phenomenon called “glycan shielding” (24). These empirical structural entities of the ORF5 significantly challenge the efficacy and the effectiveness of current vaccines and the development of novel vaccines.

Currently, there are several modified live-attenuated vaccines (MLVs) developed for PRRSV-2 that are commercially available worldwide (25). Ingelvac PRRS MLV (Boehringer Ingelheim Vetmedica, Inc.) was the first vaccine developed for PRRSV-2 and has been widely used for more than 20 years (25). MLVs are capable of limiting the incidence and severity of clinical disease, duration of viremia, and virus shedding (26); however, they have posed some challenges, such as the inability of providing complete immunity, the inefficiency of producing cross-protection against the heterologous-virulent forms, and potential recombination events between vaccine (MLVs) and wild-type strains of PRRSV-2 (27–30). Further, the Ingelvac PRRS MLV has reported a tendency of spreading the MLV strain in swine herds and reverting to higher virulent forms (31, 32). Hence, a mixture of PRRSV-2 wild-type, vaccine type (e.g., MLV-like), and recombinant strains could be circulating and persisting among pigs, further escalating the viral genetic and antigenic diversity (28). Therefore, it is essential to develop novel vaccines (e.g., multivalent vaccines) with improved safety and efficacy, which requires a comprehensive understanding of the differences in virus biology, evolution, and virus–host interaction between heterologous PRRSV strains. Molecular evolution studies provide a profound knowledge of the disparities in viral adaptation and persistence mechanisms. Thus, we aimed to investigate the genetic diversity of PRRSV-2 strains circulating among two porcine production systems (>300 farms) in the midwestern United States from 2001 to 2020 and the underlying evolutionary processes among the heterologous PRRSV-2 sequences at the molecular level.

## RESULTS

### Classification of PRRSV-2 sequences using phylogenetic analysis.

**FIG 1:**
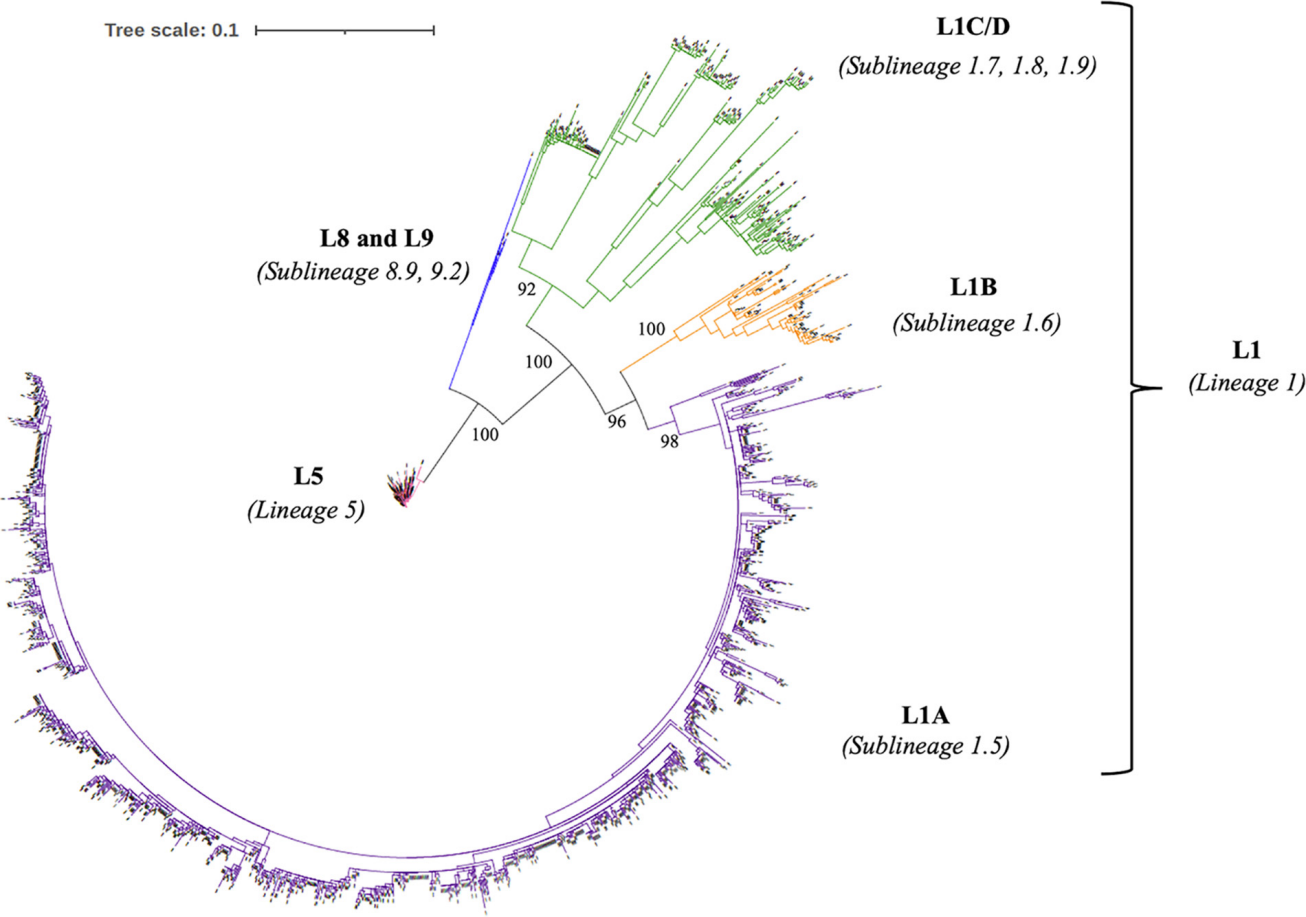
Maximum likelihood-based phylogenetic tree of 1,936 ORF5 sequences (rooted with the ORF5 sequence of Ingelvac PRRS MLV strain). The bootstraps values of the major clades are reported at each node.

This study included 1,936 ORF5 sequences of PRRSV-2 field strains that were classified using 70 global reference sequences (Table S1) (11, 33). According to the phylogenetic tree, all the PRRSV-2 sequences were classified into one of four lineages: lineage 5 (L5; *n* = 438, 22.6%), lineage 1 (L1; *n* = 1,493, 77.1%), lineage 8 (L8; *n* = 4, 0.2%), or lineage 9 (L9; *n* = 1, 0.1%) (Fig. 1, Fig. S1). The L1 strains were further classified into one of three sublineages: L1A (*n* = 1,225, 63.3%), L1B (*n* = 69, 3.6%), and L1C/D (*n* = 199, 10.3%), according to the global PRRSV classification systems (11, 33). We identified that all the L5 sequences (*n* = 438, 22.6%) were Ingelvac PRRS MLV-like while all L8 sequences (*n* = 4, 0.2%) were Fostera MLV-like using <5% nucleotide identity as a cutoff for classification of vaccine-like sequences. All the L1 sequences (*n* = 1,493, 77.1%) and the L9 sequence (*n* = 1, 0.1%) were considered wild-types based on the <5% nucleotide identity cutoff. We excluded L8 and L9 sequences from further analysis due to their small sample sizes (< 0.5%).

### Epidemiological characterization of PRRSV-2.

**FIG 2:**
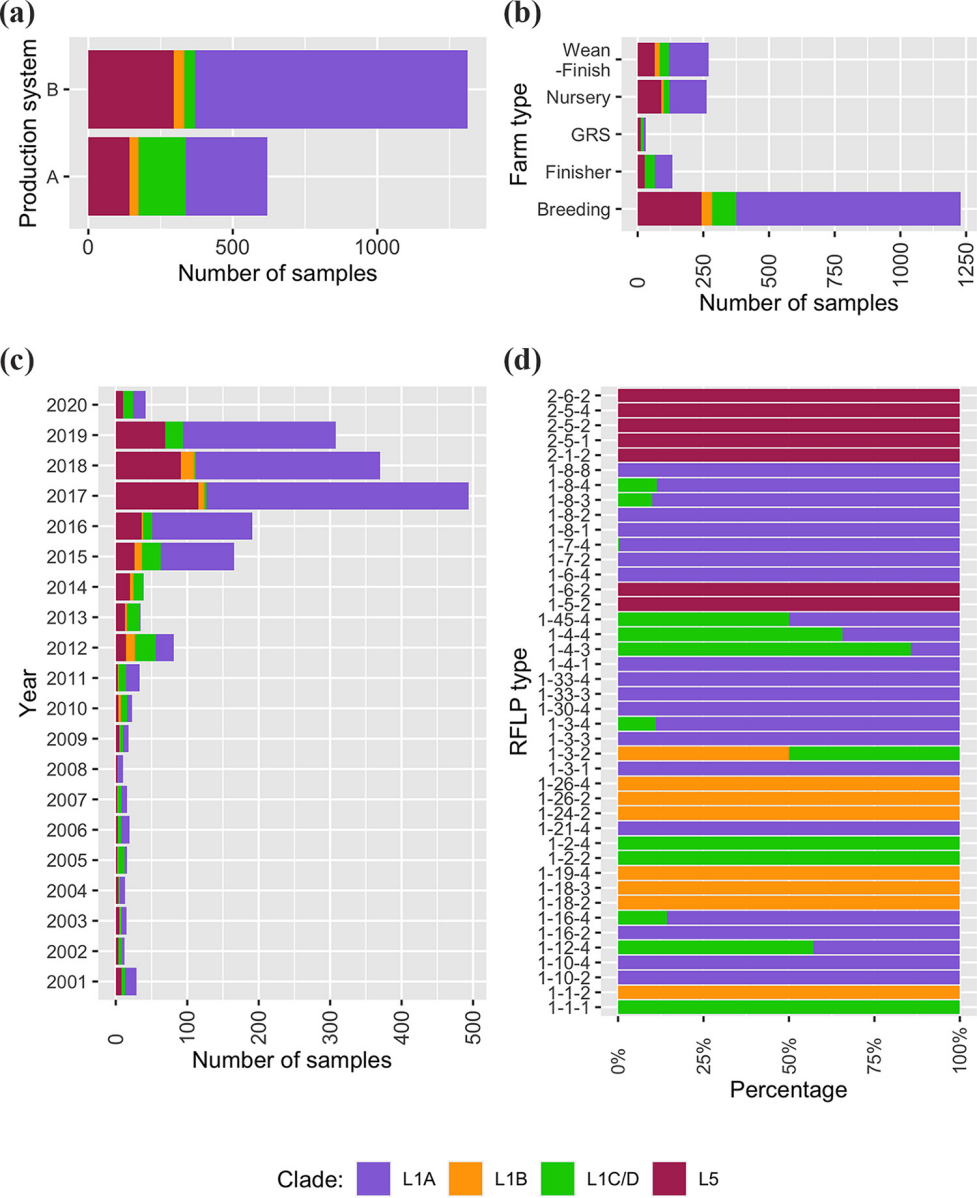
Distribution of major phylogenetic clades among (a) production systems; (b) farm types; (c) RFLP types; and (d) year. (GRS: isolation/growing replacement stocks).

PRRSV-2 sequences were mainly determined from serum (*n* = 975, 50.5%) and oral fluid (*n* = 716, 37.1%) samples. Two thirds of the field samples were obtained from production system B (*n* = 1,312, 67.9%), while the rest was collected from production system A. There was a similar percentage of L5 sequences from the production systems A and B: 142 sequences from A and 296 sequences from B, representing ~23% of strains detected in each production system. However, the proportions of L1A, L1B, and L1C/D sequences were notably different between the production systems (Fig. 2a). Especially, L1C/D sequences were more abundant in production system A than in production system B. Samples were primarily obtained from breeding (*n* = 1,230, 63.7%), followed by wean to finish (*n* = 271, 14.0%), and nursery (*n* = 263, 13.6%) herds. Nevertheless, the proportions of four clades (L5, L1A, L1B, and L1C/D) were dissimilar across the five farm types (breeding, wean to finish, nursery, finisher, and isolation/growing replacement herds). L1A was the most prevalent clade regardless of the production system or farm type, while L1B was only found in breeding, wean to finish, and nursery herds (Fig. 2b). Both L5 and L1 strains were observed consistently throughout the study period (Fig. 2c). L1A sequences were predominant in most years, although very low percentages were observed in 2013 (*n* = 1, 2.9%) and 2014 (*n* = 1, 2.6%).

We identified 35 RFLP patterns in the L1 lineage, where the most common patterns were 1-8-4 (*n* = 888, 59.5%), followed by 1-7-4 (*n* = 298, 20.0%), and 1-4-4 (*n* = 102, 6.8%). Among them, 1-8-4 (*n* = 788, 88.7%) and 1-7-4 (*n* = 297, 99.7%) were detected most consistently in the L1A sublineage, whereas 65.7% (*n* = 67) of the sequences with an RFLP 1-4-4 pattern belonged to the L1C/D. The predominant RFLP patterns of the L1B sequences were 1-1-2 (*n* = 30, 43.5%) and 1-26-2 (*n* = 28, 40.6%). Several unique RFLP patterns were identified in the L5 lineage: 1-5-2, 1-6-2, 2-1-2, 2-5-1, 2-5-2, 2-5-4, and 2–6-2, and the most common RFLP pattern was 2-5-2, which represented 93.6% (*n* = 410) of L5 sequences (Fig. 2d).

### Genetic diversity of ORF5 in PRRSV-2.

The pairwise nucleotide identity ranged from 96.3% to 100% among the L5 sequences and 83.3% to 100% in the L1 sequences (Table 1). L1C/D showed the lowest percent identities and similarities in both nucleotide and amino acid sequences of the ORF5. L1B had the highest nucleotide percent identity while L1A had the highest amino acid identity and similarity among L1 sequences. A total of 140 out of 200 sites (70%) of the predicted amino acid sequence of ORF5 were conserved across the sequences in L5 and L1B, whereas the L1C/D (110 sites; 55%) and L1A (86 sites; 43%) sublineages had relatively lower number of conserved sites.

**TABLE 1:** Pairwise nucleotide and amino acid percent identity and similarity within the L5 lineage and L1 sublineages

Lineage/sublineage	Nucleotide	Amino acid	
	Percent identity	Percent identity^*a*^	Percent similarity^*b*^
L5	96.3 to 100	92.5 to 100	94.5 to 100
L1A	88.3 to 100	87.5 to 100	91.0 to 100
L1B	90.5 to 100	83.5 to 100	87.5 to 100
L1C/D	83.0 to 100	80.5 to 100	85.5 to 100

a Amino acid sequence identity was evaluated by the matching residues.

b Amino acid sequence similarity was evaluated by the standard residue classes (GAVLI, FYW, CM, ST, KRH, DENQ, and P).

The most variable amino acid positions were 33, 58, and/or 102 in all four clades (L5, L1A, L1B, and L1C/D). L1A had 10 different amino acid residues at positions 33 and 58, where the predominant amino acids were asparagine (*n* = 896, 73.1%) and lysine (*n* = 930, 75.9%), respectively. In L1B, both 58 and 102 positions had six different amino acid residues; lysine (*n* = 28, 40.6%) and arginine (*n* = 23, 33.3%) were frequent at position 58, and serine (*n* = 33, 47.8%) and tyrosine (*n* = 25, 36.2%) were predominant at position 102. In L1C/D, positions 58, 33, and 102 had nine, eight, and seven different amino acid residues, respectively. Among them, asparagine was prevalent at position 58 (*n* = 121, 60.8%), serine (*n* = 85, 42.7%) and asparagine (*n* = 65, 32.7%) at position 33, and tyrosine (*n* = 148, 74.4%) at position 102. The most variable amino acid positions in the L5 strains were 33 and 58, where seven different amino acid residues were present at each position. The most prevalent amino acid at 33 and 58 positions was asparagine (*n* = 397, 90.6% and *n* = 402, 91.8%, respectively).

We compared amino acid substitutions of L5 strains with the Ingelvac PRRS MLV and VR2332 (the parental strain of Ingelvac PRRS MLV). Most L5 sequences had adenine at the 38th position (*n* = 419, 95.7%), similar to the vaccine strain, and the 451st position (*n* = 312, 71.2%) similar to VR2332. Although the amino acid at position 13 in the majority of L5 strains showed similarity to the vaccine strain, position 151 had six different amino acid residues, in which isoleucine (*n* = 151, 34.5%), arginine (*n* = 122, 27.9%), and glycine (*n* = 107, 24.4%) were dominant (Table 2). However, most L1 strains had lysine (*n* = 1,216, 81.4%) or asparagine (*n* = 228, 15.3%) at the 151st position. Unlike the L5 strains, L1 strains had a lower percentage of arginine (*n* = 40, 2.7%) and an absence of glycine at position 151. Nevertheless, similar proportions of amino acids at the 13th position were identified in both L5 (glutamine: *n* = 419, 95.7% and arginine: *n* = 18, 4.1%) and L1 (glutamine: *n* = 1471, 98.5% and arginine: *n* = 11, 0.7%) genetic lineages.

**TABLE 2:** Comparison of amino acids at the 13th and 151st positions of L5 and L1 strains with reference to the Ingelvac PRRS MLV strain and VR2332 (parental strain of Ingelvac PRRS MLV)

Site	Amino acid	Percent of L5 strains	Percent of L1 strains
13	Glutamine (vaccine-like)	95.7	98.5
	Arginine (VR2332-like)	4.1	0.7
	Histidine	0	0.6
151	Isoleucine	34.5	0
	Arginine (VR2332-like)	27.9	2.7
	Glycine (vaccine-like)	24.4	0
	Lysine	4.8	81.4
	Threonine	2.5	0.3
	Valine	2.3	0
	Asparagine	0	15.3
	Glutamic acid	0	0.1
	Glutamine	0	0.1

### N-glycosylation site prediction in GP5.

**FIG 3:**
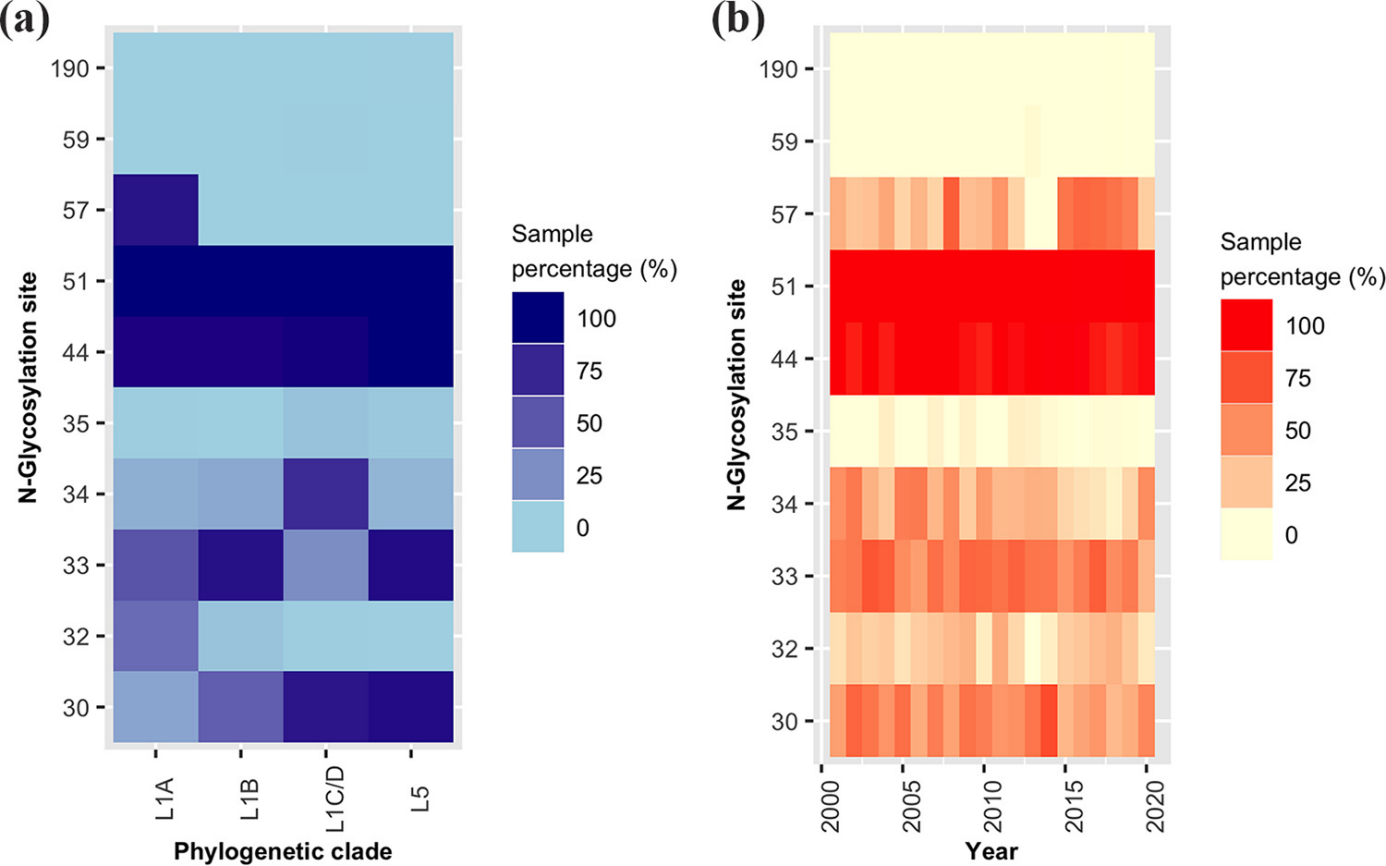
Distribution of N-glycosylation sites among (a) phylogenetic clades and (b) sampling year.

We observed 10 putative N-glycosylation sites in GP5 among the PRRSV-2 sequences (Fig. 3). Each strain had at least two N-glycosylation sites. Most sequences (*n* = 1,801, 93.3%) were N-glycosylated at positions 44 and 51, irrespective of the phylogenetic clades. Nevertheless, N-glycosylation at positions 59 and 190 were predicted only in a few sequences in the L1C/D (*n* = 10) and L5 (*n* = 1), respectively, while position 32 (except in the L1A) and 35 were infrequent N-glycosylation sites among all clades (L5, L1A, L1B, and L1C/D). The majority of L5 sequences (*n* = 386, 88.1%) had N-glycosylation at positions 30 and 33, similar to the L1C/D (*n* = 166, 83.4%) and L1B (*n* = 59, 85.5%), respectively. Site 34 was predicted for N-glycosylation mostly in L1C/D, while site 57 was exclusive to the sequences in the L1A sublineage. Position 44 in most of the sequences (*n* = 1,806, 93.5%) and positions 30, 33, 34, 35, 51, 57, and 59 in a few sequences (1 to 142 sequences, 0.1% to 7.4%) showed high specificity for N-glycosylation.

N-glycosylation positions 30, 44, 51, and 190 were conserved in most amino acid sequences with asparagine, except in site 190, where all the sequences (except one) had threonine. Site 34 was highly variable in most lineages and sublineages. However, L1 amino acid sequences had mainly serine, asparagine, or glycine, while the majority of the L5 amino acid sequences had aspartic acid (*n* = 332, 75.8%) at position 34. N-glycosylation sites: 32 and 33 had either asparagine or serine in all four clades (L5, L1A, L1B, and L1C/D). Sites 32, 34, and 57 lost the glycosylation potentials during 2010 to 2014, 2015 to 2019, and 2013 to 2014, respectively.

### Selection pressure analysis.

We found nine and 19 positions in the GP5 sequence of L5 and L1, respectively, with significant positive selection by fixed effects likelihood (FEL), internal fixed effects likelihood (IFEL), and fast, unconstrained Bayesian approximation (FUBAR) methods (Table 3). Positions 3, 25, 26, 32, 34, and 35 showed significant local positive selection in both L5 and L1 lineages, whereas positions 13, 151, and 200 were exclusive to the L5 sequences for positive selection.

**TABLE 3:** Significant amino acid positions that undergo positive selection in L5 and L1 lineages, based on IFEL, FEL, and FUBAR methods

Lineage	Predicted biological regions of GP5	Codon positions^*a*^
L5	Signal peptide	3, 13, 25, 26
	Ectodomain	32, 34, 35
	Transmembrane	ND^*b*^
	Endodomain	151, 200
L1	Signal peptide	3, 11, 15, 16, 17, 19, 25, 26
	Ectodomain	32, 33, 34, 35, 41, 58, 59
	Transmembrane	98, 102, 104
	Endodomain	189

a Only the sites that were significant in all three methods are shown here (significant positive selection sites in each method are shown in Table S2).

b ND, not detected.

**FIG 4:**
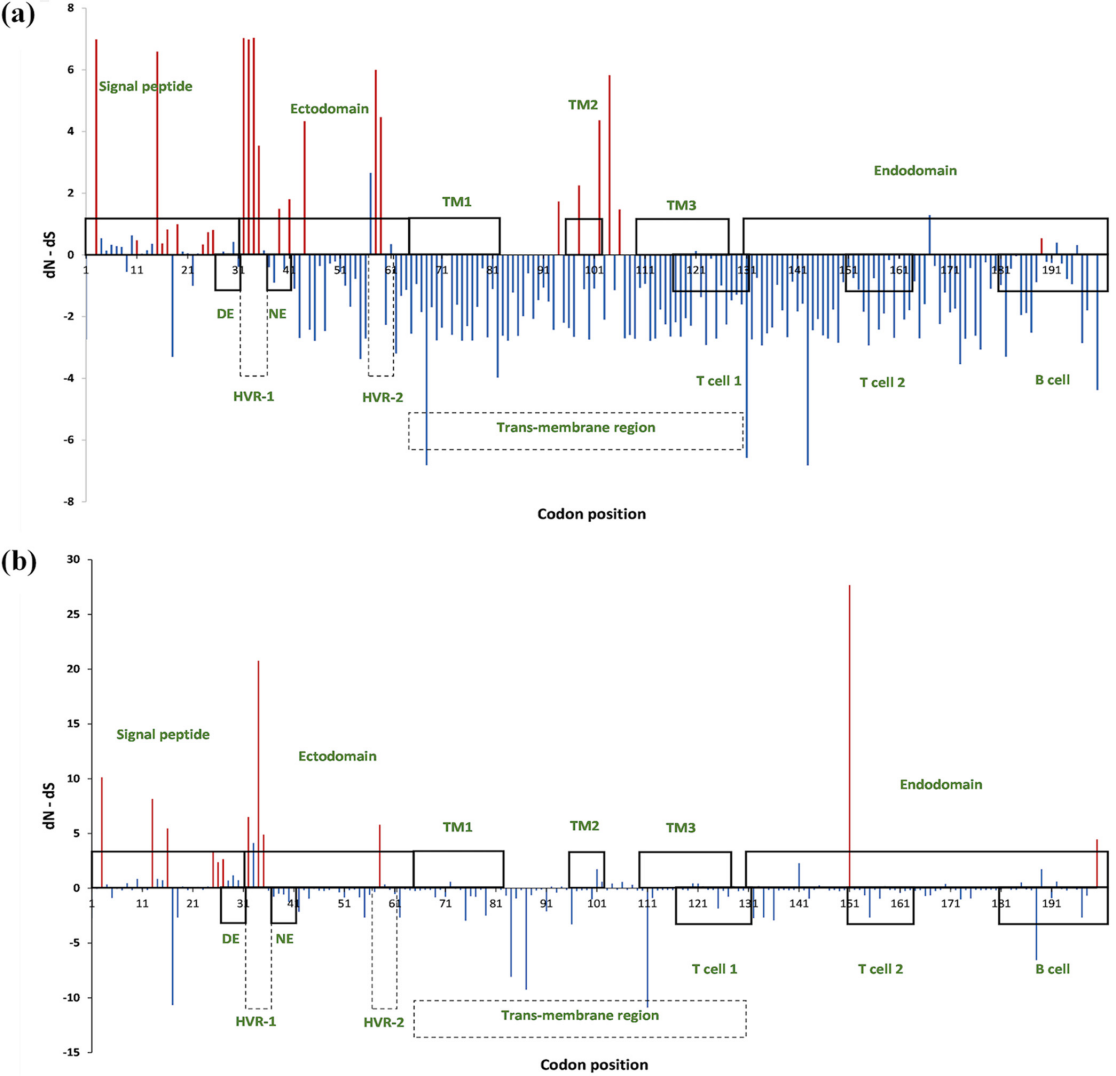
Codon positions under selective pressure in the (a) L1 lineage and (b) L5 lineage. The upper rectangles indicate the signal peptide, ectodomain, transmembrane (TM) regions (TM1, TM2, and TM3), and endodomain, whereas the lower rectangles indicate the decoy epitope (DE), neutralizing epitope (NE), hypervariable regions (HVR-1 and HVR-2), two T cell epitopes and the 3rd B cell epitope (19–21, 63). The red lines indicate significant sites under positive selection. *y* axis represents the *dN-dS* values under the FUBAR method.

L5 lineage exhibited higher *dN-dS* values (the difference between *dN* and *dS* rates) at the majority of the significant positive selection sites compared to those in the L1 lineage in any method (Fig. 4a and b). Among them, 34 and 151 codon positions in the L5 sequences showed prominent *dN-dS* values. Most of the positive selection sites in both L5 and L1 lineages were in the signal peptide and ectodomain regions in the encoded GP5 protein (Table 3, Fig. 4a and b). Only two positive selection sites (189 and 196 positions based on FUBAR) were found in the endodomain in the L1 sequences, while L5 sequences had two such sites at 151 and 200 positions in the endodomain (Fig. 4a and b).

**FIG 5:**
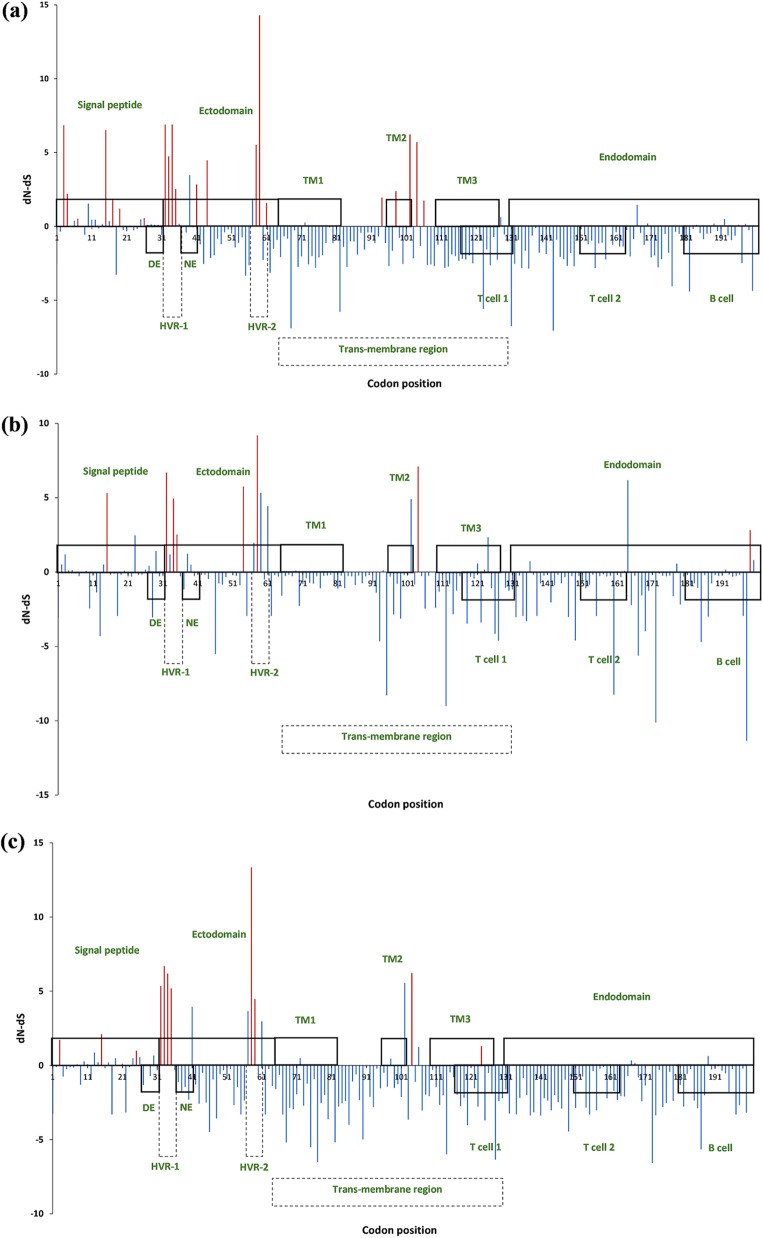
Codon positions under selective pressure among the three L1 sublineages. (a) L1A sublineage; (b) L1B sublineage; and (c) L1C/D sublineage. The upper rectangles indicate the signal peptide, ectodomain, transmembrane (TM) regions (TM1, TM2, and TM3), and endodomain, whereas the lower rectangles indicate the decoy epitope (DE), neutralizing epitope (NE), hypervariable regions (HVR-1 and HVR-2), two T cell epitopes and the 3rd B cell epitope (19–21, 63). The red lines indicate significant sites under positive selection. *y* axis represents the *dN-dS* values under the FUBAR method.

Significant positive selection sites in L1A, L1B, and L1C/D strains were also estimated (Fig. 5). Ectodomain of the putative GP5 in all three L1 sublineages had the highest number of positive selection sites (L1A: nine out of 21; L1B: five out of eight; L1C/D: six out of 11), as opposed to the L5 lineage, where the signal peptide had more positive selection sites (six out of 12) (Fig. 4 and 5). A total of 40 significant positive selection sites were present in the L5 lineage and three L1 sublineages (Fig. S2), while only four positive selection sites: 32, 34, 35 (FUBAR), and 58 (IFEL, FUBAR), were common among the four clades (L5, L1A, L1B, and L1C/D). Positions 54 (by FUBAR) and 199 were unique to the L1B, while positions 124 and 189 (IFEL, FEL) were present only in L1C/D. L1A had many unique codon positions that undergo positive selection compared with the other L1 sublineages and L5 lineage. Conversely, L1B had fewer significant positive selection sites.

Among the significant positive selection sites, only a few positions displayed a notable difference in the leading amino acid among L1 and L5 strains: positions 3 (glycine to glutamic acid), 16 (phenylalanine to serine), 27 (alanine to valine), 94 (isoleucine to valine), 151 (lysine to isoleucine, arginine, or glycine), and 189 (valine to isoleucine) as illustrated in Table S3. Most of the significant positive selection sites in L1A sublineage (16 out of 21 sites) and L5 lineage (eight out of 12 sites) had the highest *dN/dS* ratios relative to the other L1 sublineages (Fig. 4 and 5; Table S4). We identified six, four, four, and five positions under very high local positive selection pressure (*dN/dS* ratio > 10) in the L1A (positions 3, 15, 32, 33, 34, and 59), L1B (positions 15, 32, 58, and 104), L1C/D (positions 32, 33, 34, and 35), and L5 (positions 3, 13, 32, 34, and 151), respectively (Fig. 4 and 5; Table S4).

Based on the global selection pressure analysis, the L5 strains showed an overall diversifying selection pressure (*dN/dS* ratio >1), while the L1 lineage and all its sublineages exhibited overall purifying selection (*dN/dS* ratio <1) as shown in Table 4. L1C/D showed the lowest *dN/dS* ratios compared with any lineage/sublineage.

**TABLE 4:** Global *dN/dS* ratio of each lineage and sublineage based on FEL, IFEL, and FUBAR methods

Lineage/sublineage	FEL	IFEL	FUBAR
L5	1.1	2.6	1
L1	0.4	0.3	0.6
L1A	0.5	0.4	0.6
L1B	0.5	0.5	0.6
L1C/D	0.3	0.3	0.5

## DISCUSSION

Since the first introduction of PRRSV into the United States in the late 1980s, PRRSV has widely spread among pigs and is continuously evolving via point mutations and genome recombination (34). The purpose of this study was to evaluate genetic characteristics of PRRSV-2 and underlying evolutionary processes. Thus, we examined the genetic diversity and molecular attributes of PRRSV-2 detected in >300 swine farms in the midwestern United States from 2001 to 2020. Most PRRSV-2 strains in our study were classified either lineage 1 or lineage 5, whereas lineages 8 and 9 were rarely identified. Interestingly, lineages 1 and 2 viruses were first originated from Canada, and not found in the United States until 1998 (35). Over time, viruses in these lineages have become dominant and largely replaced the original local virus populations (lineages 6 to 9) in the United States (10). Based on our findings, L1A (sublineage 1.5) and L5 (sublineage 5.1) strains were prominent in both production systems, farm type, and sampling year during the study period (except in 2013 and 2014). Therefore, we acknowledged the significance of L1A and L5 strains within the studied swine population. Conversely, L1B and L1C/D (sublineage 1.6 to 1.9) strains were present in relatively low percentages.

Previous studies reported a higher virulence and potential of shedding via aerosols for lineage 1 and 2 strains, especially for strains with RFLP 1-8-4 and 1-22-2 patterns, compared with the endemic lineages (lineages 6 to 9) (35). RFLP 1–7-4 strains were previously reported causing intense abortion storms in sow herds in the United States (36). Based on our study, most of the L1 strains were either RFLP 1-8-4 or 1-7-4 type. Strains with RFLP 1-8-4 type were grouped into the sublineage 1.9 (MN184-like) in previous studies (33), yet most of our strains that had RFLP 1-8-4 type belonged to the sublineage 1.5 (L1A). It signifies the limitations of using RFLP typing for sublineage classification within PRRSV-2 lineages. Previous studies reported instability of RFLP patterns during sequential pig-to-pig passages and higher genetic diversity among field strains with the same RFLP pattern (4, 37). Nevertheless, L5 strains were found to have unique RFLP patterns that were not encountered among L1 strains in our study.

Viral protein N-glycosylation is a complex post-translational modification that affects several biological processes in viruses, including receptor interactions, viral entry, protein folding, immune evasion, and pathogenesis (38, 39). Hypoglycosylated GP5 produces a significantly higher neutralizing antibody response in PRRSV-infected pigs (24). Most of our strains were glycosylated at least at the conserved N-glycosylation sites at N44 and N51, similar to the previous studies (24). According to Ansari et al., adding glycan at site 44 in PRRSV-2 GP5 protein is vital for infectious progeny production and recovery of infectious virus (24), which denotes N44 as one of the most crucial amino acid for viral infectivity. Glycans at sites N34 and N51 potentially act as a viral protein shield against host antibody neutralization (glycan shielding) (24). Therefore, the majority of our strains may have the capability to evade the host immune system, promoting persistent infections (40). Site N34, on the other hand, was mainly glycosylated in the L1C/D strains in our study. We identified an excessive positive selective pressure at site 34 in L1A, L1C/D, and L5. The tremendous variability at site 34 may cause this disparity in acquisition or loss of glycosylation ability among heterologous strains in this study.

According to previous studies, the HVR-1 at the boundary between signal peptide and ectodomain is often rich in serine and asparagine codons, thus, prone to the addition or loss of N-glycosylation sites (40). We recognized five potential N-glycosylation sites (30, 32, 33, 34, and 35) in the HVR-1 (between the putative DE and NE epitopes) of the putative GP5 in most strains, similar to previous studies (41), and these sites may boost the signal peptide cleavage, as described by Thaa et al. (40). Two likely cleavage sites were previously specified in GP5 of PRRSV-2 (reference strain VR-2332): site 1 (between alanine at position 26 and valine at position 27) and site 2 (between alanine at position 31 and serine at position 32) (40). Cleavage at site one preserves DE, reducing the host antibody response against the nearby NE, which promotes the persistent infections of PRRSV (19, 40). However, cleaving at site two removes the DE in the mature GP5 protein chain (19). Therefore, clade-specific acquisition or loss of N-glycosylation sites in this region may diversify the nature of the virus-host interaction. Further, we identified a unique glycosylation site (site 57) in the HVR-2 in L1A strains (*n* = 1,036, 84.6% strains). It is essential to investigate the biological impact of glycosylation at site 57 on virus-host interaction and evolutionary trajectories of the L1A sublineage. However, this N-glycosylation site was rarely identified in 2013 to 2014, which is explained by the low percentages of L1A strains detected during this period. According to previous studies, there was significantly less PRRS reported within many production systems in the United States in 2013 to 2014, and there was a delay in the onset of the epidemic (42). In 2013 to 2014, porcine epidemic diarrhea virus (PEDV), which causes diarrhea and vomiting in adult pigs and high mortality in neonatal piglets, was introduced into the United States swine herds, modifying epidemiological conditions of the United States. Therefore, the reduction of L1A in 2013 to 2014 could be due to the improved biosecurity measures aimed at preventing PED transmission which may have reduced PRRS incidence, fewer diagnostic tests on PRRSV, and reduced animal movements between farms, etc. For example, recent epidemiological studies suggested that the occurrence of PRRSV-2 lineages/sublineages was predominantly influenced by animal movements than the spatial proximity of farms. More importantly, secondary farm contacts (via animal movements) with L1A-positive farms (RFLP 1-7-4 like) have been identified as a risk factor for L1A occurrence on a farm (43).

Recent studies suggested that the perpetual evolutionary arms race between host and viruses due to host immunity can produce a robust adaptive evolution on PRRSV strains, especially in sites that encode for epitopes recognized by the host immune system (40). Thus, the positive selection at such sites, if present, suggests that it originated from the host immune response. Interestingly, we discovered positive selection at the putative epitopes only in L1A (at NE) and L5 (at DE), which may enhance the adaptability of the L1A and L5 strains among swine herds. The hydrophobic transmembrane region of the putative GP5 is presumed to span the endoplasmic reticulum (ER) membrane three times, producing an outward protrusion at the end of the TM2 region in the GP5 (40). We identified two to three significant positive selection sites at this region (102, 104, and/or 106) among the L1 sublineages. Fan et al. revealed an ability to enhance PRRSV evasion of neutralizing antibodies *in vitro* by site-directed mutagenesis of the amino acid residues at 102 and 104 (44). Additionally, having many positive selection sites in the signal peptide region of GP5 in both L5 and L1 strains may affect the transfer of GP5 to the ER membrane.

In our study, 22.6% (*n* = 438) of the sequences belonged to L5 and were closely related to the Ingelvac PRRS MLV, inferring the importance of the vaccine in shaping the current PRRSV diversity in the United States. However, it is not yet clear whether these L5 strains were originated from the Ingelvac PRRS MLV or its parental wild-type strain (VR2332) circulating in the field. The intralineage diversity within the L5 sequences was notably low compared with the other sublineages, suggesting that at least a large proportion of the L5 strains found in this study were indeed vaccine-related. It was of interest to explore whether the changes in the Ingelvac PRRS MLV strain were due to the random mutation or undergoing adaptive selection pressure. Elevated *dN-dS* values in L5 strains (compared with those in L1 strains) at most positive selection sites support the idea of positive selection pressure. The unique positive selection sites (13, 151, 200) identified in the L5 lineage in our study potentially favor the evolutionary fitness of the strains. The higher global *dN/dS* ratio provides additional evidence for the ongoing evolution and likely higher adaptation of L5 strains. Previous studies explained that arginine at position 151 is more common and stable, as in the VR2332 (45, 46), whereas glycine at position 151 was a vaccine marker (47). The highest *dN-dS* value at position 151 may explain the previously documented instability of the Ingelvac PRRS MLV and reversion to virulent forms (31, 32). However, according to our study, the most common amino acid at position 151 in L5 strains was isoleucine, followed by arginine and glycine. On the other hand, glutamine at position 13 in the vaccine strain is considered a conservative mutation located in the presumed signal peptide (48). Mutations in this site may influence the function of the signal peptide and likely interfere with the GP5 transport to the ER membrane. Continuous spread and evolution of these L5 strains could potentially develop new variants causing novel outbreaks.

In summary, the present study provides valued insights into the roles of genetic variation/mutation in driving PRRSV-2 evolution within the two swine production systems in the United States. However, there are a few limitations to this study. Our study utilized only the polymorphisms in ORF5 to make inferences in genetic diversity, molecular, and evolutionary characteristics of PRRSV-2 strains. Although ORF5 is highly variable and commonly studied, the cumulative information of multiple ORFs or whole-genome sequences of PRRSV will increase the generalizability of the results. Additionally, we assessed only a limited number of sequences (< 2,000) and the PRRSV-2 genetic data in our study represented only two production systems, thus we cannot generalize our results to the entire swine population in the United States. Previous studies disclosed the importance of animal movements, the structure of the swine shipment network, and the connectivity between production sites for the PRRSV-2 transmission within swine herds (43, 49). Therefore, variation in PRRSV-2 lineage/sublineage prevalence and emergence of novel strains could be a result of an introduction of new strains from other areas, in addition to evolutionary or recombination processes of existing strains, which challenges the PRRSV-2 epidemiology in the studied region. Therefore, further studies incorporating multiple swine production systems from a wider geographical area are recommended.

Continuous emergence of novel PRRSV-2 variants demands updating and expanding the existing PRRSV-2 lineage/sublineage classification systems. In this study, we used lineage/sublineage classification systems introduced by Paploski et al. and Shi et al. to identify and label our PRRSV-2 strains (11, 33). Based on the bootstrap criteria used in this study, L1C and L1D (sublineages 1.7, 1.8, and 1.9) were clustered into a single clade (L1C/D). It explained the higher intra-clade diversity within L1C/D compared with the L5 lineage and other L1 sublineages. Paploski et al. expanded L1 lineage further creating nine L1 sublineages (L1A-L1C, L1Dalfa, L1Dbeta, L1E-L1H) (12). Based on that, the L1C/D clade was further divided into multiple sublineages (L1C, L1Dbeta, L1F, and L1H). Nevertheless, the increment of the intra-clade diversity within L1C/D is lower than expected in terms of the pairwise amino acid percent identity and similarity, and the presence of conserved sites (55% sites in the deduced GP5), which may be due to the high genetic similarity between these sublineages. Finally, we did not evaluate the clinical impact of the heterologous strains in this study. Association studies correlating the phenotypic characteristics are valuable to identify the expression levels of the genomic variations. Therefore, future studies should be conducted to comprehend the biological and clinical role of the heterologous strains in parallel to their evolutionary characterization.

## MATERIALS AND METHODS

### Sample collection and preparation.

We used genetic information of the ORF5 of PRRSV-2, detected from >300 farms managed by two pork production systems in the midwestern United States. A total of 1,936 field samples (blood, oral fluids, serum, tissue samples, swabs, testicle, and tail processing fluids) were collected from the infected pigs from 2001 to 2020 and processed for RNA extraction and virus isolation. The viral ORF5 was amplified by reverse transcription-PCR (RT-PCR) and sequenced at veterinary diagnostic laboratories. According to the previous research of global PRRSV-2 classification (33, 50), 70 global reference sequences of PRRSV-2 (603 bp) were collected from the NCBI GenBank database (Table S1). A total of 2,006 PRRSV-2 ORF5 gene sequences with 603 bp were aligned for the phylogenetic analysis.

### Classification of PRRSV-2 using phylogenetic analysis.

ORF5 sequences were aligned using the MUltiple Sequence Comparison by Log-Expectation (MUSCLE) algorithm in AliView (Version 1.26) (51). Phylogenetic analysis was performed on aligned ORF5 sequences by the maximum likelihood method using IQ-TREE (Version 2.1.1) in CIPRES Science Gateway (Version 3.3) (https://www.phylo.org/portal2/login!input.action) (52, 53). The best-fit nucleotide substitution model was determined by ModelFinder, according to the Bayesian information criterion (BIC) (54). Therefore, we selected the best-fit model combining two nucleotide substitution models based on codon position scheme (1st and 2nd + 3rd nucleotide positions): K3Pu+F+R5 for 1st and 2nd codon positions and SYM+I+G4 for 3rd codon position (52). The best-fit model was extended to accommodate the among-site rate variation and the among-site process heterogeneity via partitioned-data approaches for the three codon positions. Bootstrap values were assessed using the ultrafast bootstrap approximation method with 5,000 replicates (55). The phylogenetic tree was visualized by the interactive Tree of Life (iTOL) tool (https://itol.embl.de/) (56). Based on the tree topology, the clades with high bootstrap values (>95%) were identified and classified according to the global PRRSV classification systems (11, 33). Epidemiological characteristics of phylogenetic clades were evaluated, based on the distribution of production systems, farm types, sampling years, and the restriction fragment length polymorphism (RFLP) patterns (57) within clades. Additionally, we assessed the pairwise nucleotide and amino acid percent identity and similarity within each phylogenetic clade using Sequence Manipulation Suite (Version 2) (https://www.bioinformatics.org/sms2/ident_sim.html) (58). Further, we compared amino acid substitutions in MLV-like strains with the Ingelvac PRRS MLV strain and VR2332 (the parental strain of Ingelvac PRRS MLV).

### Glycosylation site prediction.

N-glycosylation sites in the putative GP5 were predicted using the NetNGlyc (1.0) (https://services.healthtech.dtu.dk/service.php?NetNGlyc-1.0) (59). The default threshold of 0.5 was applied to predict the potential N-glycosylation sites, whereas 0.75 and 0.9 threshold levels were used to determine the N-glycosylation sites with high confidence. The results were summarized based on the phylogenetic clades identified in our study.

### Local and global genetic selection pressure estimation.

Local genetic selection pressure on PRRSV-2 ORF5 sequences within each phylogenic clade was evaluated by the site-specific *dN/dS* ratio, which compares the rate of substitutions at non-synonymous sites (*dN*) to the rate of substitutions at synonymous sites (*dS*). Selection pressure was estimated using FEL, IFEL, and FUBAR algorithms in Hyphy (Version 2.5.17) (https://github.com/veg/hyphy/releases/tag/2.5.17) (60–62). GP5 sites with *dN/dS* ratios higher than one at *P*-value ≤0.1 significance level by FEL and IFEL methods and at posterior probability ≥0.9 by the FUBAR method considered under positive selection. Because each approach exploits a different algorithm for prediction, we selected only the sites undergoing local positive selection predicted by all three algorithms. Global genetic selection pressure was determined, averaging the local *dN/dS* ratio over the GP5 using all three algorithms.

### Data availability.

The data that support the findings of this study are available upon reasonable request to the corresponding author (B.M.). The data are not publicly available due to proprietary and privacy restrictions from third parties (companies, veterinarians, diagnostic laboratories, etc.).
