# Supplementary material for: Molecular Evolution of Porcine Reproductive and Respiratory Syndrome Virus Field Strains from Two Swine Production Systems in the Midwestern United States from 2001 to 2020
Source: Microbiol Spectr. 2022 May 2;10(3):e02634-21. doi: 10.1128/spectrum.02634-21 (PMC9241855; doi:10.1128/spectrum.02634-21)
Supplement: SUPPLEMENTAL FILE 2 — Supplemental material. Download spectrum.02634-21-s002.pdf, PDF file, 0.3 MB [file spectrum.02634-21-s002.pdf]

SUPPLEMENTARY DOCUMENT

FIGURES:

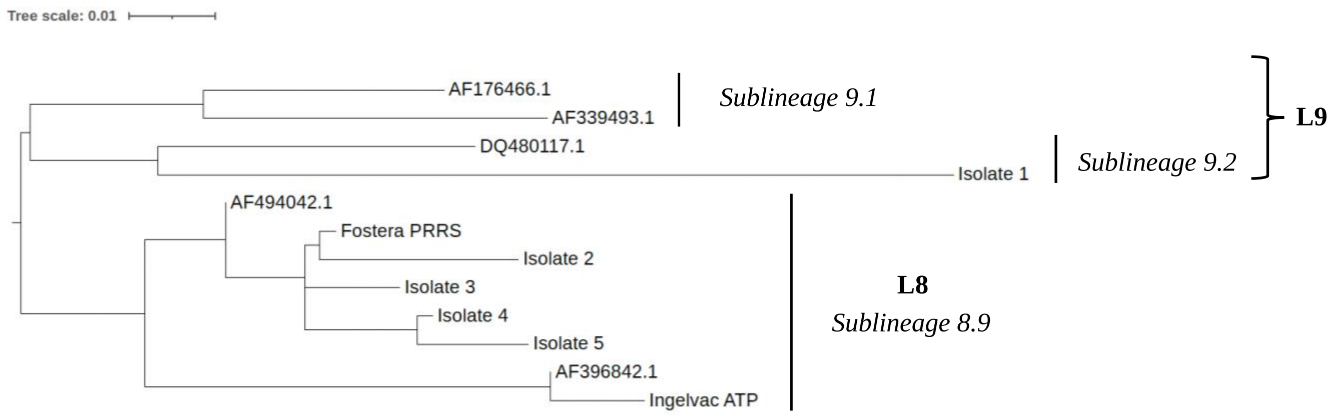

FIG S1 Maximum likelihood-based phylogenetic tree of the five ORF5 sequences that belonged to the L8 and L9 lineages, according to the global PRRSV lineage classification systems. The five ORF5 sequences are represented by Isolate 1-5.

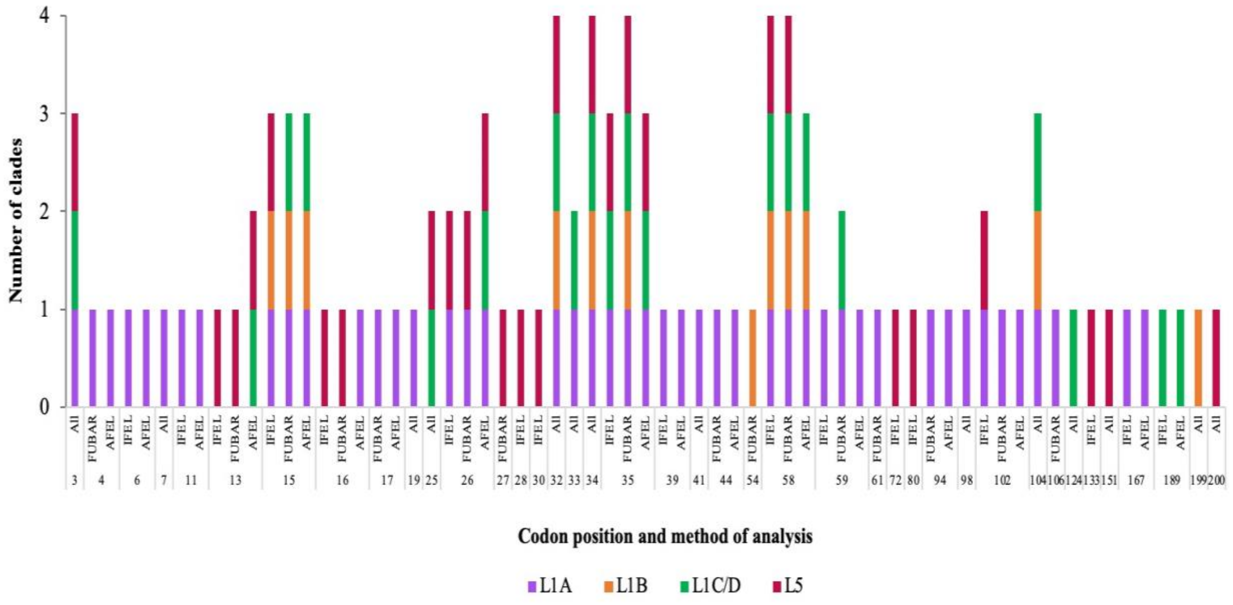

FIG S2 Significant positive selection sites identified by FUBAR, FEL, and IFEL methods. “All” on the x-axis indicates all methods.

TABLES:

TABLE S1 PRRSV reference sequences used in this study.

No.	Virus strain	Accession no.	Lineage/ Sublineage[a]	New labels[b]	No.	Virus strain	Accession no.	Lineage/ Sublineage[a]	New labels[b]
1	98-3298	DQ306877	1.1	-	36	Aichi N20	AB175715	7	L7
2	98-3403	DQ306878	1.1	-	37	Neb-1	EU755263	7	L7
3	99-3584	DQ306879	1.1	-	38	CH-1a	AY032626	8.1	L8
4	PRRSV-2_Hungary_102_2012	KM514315	1.2	-	39	HH08	JX679179	8.1	L8
5	K05-0050	JQ656133	1.2	-	40	HBJM2	EU399826	8.2	L8
6	2002-1347	EU556211	1.5	L1A	41	HBSZ	EU399825	8.2	L8
7	FJ0908	MK202794	1.5	L1A	42	JXA1	EF112445	8.3	L8
8	OH/2014/ISU-7	MF326994	1.5	L1A	43	JXwn06	EF641008	8.3	L8
9	PRRSV2/Michigan/XW074/2011	KP283440	1.6	L1B	44	TJ	EU860248	8.3	L8
10	NADC31	JN660150	1.6	L1B	45	GXLSN06\'962012	KC618172	8.4	L8
11	PRRSV2/Indiana/XW054/2012	KP283421	1.6	L1B	46	JXZX2	EU399849	8.4	L8
12	2001-18565	EU556201	1.7	L1C/D	47	AHW01	EU399828	8.5	L8
13	PRRSV2/USA/AP244/2010	KT902607	1.7	L1C/D	48	HeN-2	FJ237419	8.5	L8
14	PRRSV2/Michigan/XW090/2011	KP283456	1.7	L1C/D	49	SDSU73	AY656993	8.5	L8
15	NADC30	JN654459	1.8	L1C/D	50	JXZX2	HQ832215	8.4	L8
16	CHsx1401	KP861625	1.8	L1C/D	51	Yunnan-08	EU819086	8.6	L8
17	HNhx	KX766379	1.8	L1C/D	52	HK1	KF287132	8.7	L8
18	MN184	EF442777	1.9	L1C/D	53	HK4	KF287134	8.7	L8
19	MN184A	DQ176019	1.9	L1C/D	54	JA-142	AF396842	8.9	L8
20	MN184C	EF488739	1.9	L1C/D	55	P129	AF494042	8.9	L8
21	03-03056	DQ480123	1.9	L1C/D	56	98-6470-1	AF339493	9.1	L9
22	2000-48305	EU556178	1.9	L1C/D	57	PRRSV44	AF176466	9.1	L9
23	PRRSV0000008659	EU758687	2	L2	58	02-01676	DQ480117	9.2	L9
24	PRRSV0000008973	EU758940	2	L2	59	98-5579-1	AF339500	9.8	L9

25	PRRSV0000031	DQ474791	2	L2	60	B1	AY318773	9.9	L9
26	FJ-1	AY881994	3	L3	61	PRRSV16	AF176438	9.9	L9
27	GD-KP	KU978619	3	L3	62	2002-5252C	EU556220	9.12	L9
28	GM2	JN662424	3	L3	63	03-07053	DQ480124	9.13	L9
29	Ibaraki08\'965	AB546113	4	L4	64	2000-54471A	EU556182	9.15	L9
30	Miyagi08\'962	AB546105	4	L4	65	Prevacent PRRS		1.7	L1
31	Miyagi08\'963	AB546106	4	L4	66	Ingelvac MLV		5	L5
32	VR-2332	AY150564	5	L5	67	Asian PRRS		8	L8
33	PA8	AH006184	5.1	L5	68	PrimePac	AF066384	7	L7
34	NADC-8	AF396833	5.2	L5	69	Ingelvac ATP	DQ988080	8	L8
35	NVSL-14	AF396839	6	L6	70	Fostera PRRS		8	L8

[a] Based on Shi M, Lam TT-Y, Hon C-C, Murtaugh MP, Davies PR, Hui RK-H, Li J, Wong LT-W, Yip C-W, Jiang J-W, Leung FC-C. 2010. Phylogeny-based evolutionary, demographical, and geographical dissection of North American type 2 porcine reproductive and respiratory syndrome viruses. J Virol 84:8700–8711.

[b] We labeled all 2006 ORF5 sequences used in our study according to the lineage/sublineage labels introduced by Paploski IAD, Corzo C, Rovira A, Murtaugh MP, Sanhueza JM, Vilalta C, Schroeder DC, VanderWaal K. 2019. Temporal dynamics of co-circulating lineages of porcine reproductive and respiratory syndrome virus. Front Microbiol 10:2486.

TABLE S2 Significant codon positions that undergo positive selection in L5 and L1 lineages based on IFEL, FEL, and FUBAR methods.

Lineage	Predicted GP5 region	FEL[a]	IFEL	FUBAR
L5	Signal peptide	**3, 13, 25, 26, 32**	3, 13, 15, 16, 25, 26, 28, 30, 32	3, 13, 16, 25, 26, 27, 32
	Ectodomain	**34, 35**	34, 35, 58	34, 35, 58
	Transmembrane		72, 80, 102, 133	
	Endodomain	**151, 200**	151, 200	151, 200
L1	Signal peptide	**3**, 4, 6, 8, **11**, **15**, **16**, **17, 19,** 24, **25, 26, 32**	3, 8, 11, 15, 16, 17, 19, 25, 26, 32	3, 11, 15, 16, 17, 19, 24, 25, 26, 32
	Ectodomain	**33**, **34**, **35**, 39, **41**, **58**, **59**	33, 34, 35, 41, 58, 59	33, 34, 35, 39, 41, 44, 58, 59
	Transmembrane	**98**, **102**, **104**	98, 102, 104	94, 98, 102, 104, 106
	Endodomain	**189**, 196	189	189

[a] The sites in bold letters were common in all three methods in both L5 and L1 lineages.

TABLE S3 Major amino acid at each positive selection site and the frequency (%) within the L1 sublineages (L1A, L1B, and L1C/D) and L5 lineage.

Site	L1A		L1B		L1C/D		L5	
	AA	%	AA	%	AA	%	AA	%
3	G	93.6	G	92.8	G	56.3	E	82.6
15	P	83.8	L	85.5	P	72.4	L	99.1
16	F	99.1	F	100.0	F	100.0	S	96.1
26	V	98.3	V	94.2	A	96.5	A	98.4
27	A	98.9	A	91.3	A	99.0	V	98.2
32	N	80.9	S	73.9	S	84.4	S	94.5
33	N	73.1	N	91.3	S	42.7	N	90.6
34	S	71.3	N	58.0	N	73.9	D	75.8
58	K	75.9	K	40.6	N	60.8	N	91.8
59	S	85.9	K	65.2	K	53.8	K	99.3
94	I	91.1	I	91.3	I	96.0	V	99.3
98	A	83.7	A	53.6	T	91.5	T	100.0
102	Y	90.8	S	47.8	Y	74.4	V	99.3

151	K	80.1	K	97.1	K	84.4	I	34.5
167	I	85.6	I	95.7	V	92.0	V	100.0
189	V	99.7	V	100.0	V	94.0	I	98.6

TABLE S4 Significant positive selection sites and the site-specific *dN/dS* ratios of the L1 sublineages (L1A, L1B, and L1C/D) and L5 lineage based on the FUBAR method.

Predicted GP5 region	L1A		L1B		L1C/D		L5	
	Site	*dN/dS*	Site	*dN/dS*	Site	*dN/dS*	Site	*dN/dS*
Signal peptide	3	26.9	15	10.5	3	3.9	3	16.4
	4	5.3			15	3.9	13	15.6
	7	3.1			25	3.7	16	5.6
	15	12.0					25	7.0
	17	4.8					26	5.6
	19	6.1					27	5.5
	26	3.7						
Ectodomain	32	31.6	32	10.9	32	14.1	32	11.2
	33	19.8	34	8.5	33	17.3	34	38.1
	34	32.4	35	5.1	34	16.8	35	9.0
	35	9.6	54	4.8	35	13.6	58	4.9
	41	4.7	58	15.8	58	7.2		
	44	2.7			59	2.7		
	58	4.5						
	59	14.6						
	61	2.5						
Transmembrane	94	3.3	104	10.9	104	6.0		
	98	5.5			124	4.3		
	102	8.0						
	104	5.0						
	106	2.9						
Endodomain			199	6.2			151	26.2
							200	9.2
